# Work Incapacity and Treatment Costs After Severe Accidents: Standard Versus Intensive Case Management in a 6-Year Randomized Controlled Trial

**DOI:** 10.1007/s10926-015-9615-0

**Published:** 2015-12-21

**Authors:** Stefan M. Scholz, Peter Andermatt, Benno L. Tobler, Dieter Spinnler

**Affiliations:** Suva (Swiss National Accident Insurance Fund), Lucerne, Switzerland

**Keywords:** Case management, Accident, Insurance, Return to work, Treatment costs

## Abstract

*Purpose* Case management is widely accepted as an effective method to support medical rehabilitation and vocational reintegration of accident victims with musculoskeletal injuries. This study investigates whether more intensive case management improves outcomes such as work incapacity and treatment costs for severely injured patients. *Methods* 8,050 patients were randomly allocated either to *standardcase management* (SCM, administered by claims specialists) or *intensive case management* (ICM, administered by case managers). These study groups differ mainly by caseload, which was approximately 100 cases in SCM and 35 in ICM. The setting is equivalent to a prospective randomized controlled trial. A 6-year follow-up period was chosen in order to encompass both short-term insurance benefits and permanent disability costs. All data were extracted from administrative insurance databases. *Results* Average work incapacity over the 6-year follow-up, including contributions from daily allowances and permanent losses from disability, was slightly but insignificantly higher under ICM than under SCM (21.6 vs. 21.3 % of pre-accident work capacity). Remaining work incapacity after 6 years of follow-up showed no difference between ICM and SCM (8.9 vs. 8.8 % of pre-accident work incapacity). Treatment costs were 43,500 Swiss Francs (CHF) in ICM compared to 39,800 in SCM (+9.4 %, *p* = 0.01). The number of care providers involved in ICM was 10.5 compared to 10.0 in ICM (+5.0 %, *p* < 0.001). *Conclusions* Contrary to expectations, ICM did not reduce work incapacity as compared to SCM, but did increase healthcare consumption and treatment costs. It is concluded that the intensity of case management alone is not sufficient to improve rehabilitation and vocational reintegration of accident victims.

## Introduction

Severe accidents have considerable occupational, economic and social consequences for the victims and their families. Return to work (RTW) is a major issue in the process of vocational reintegration of these patients, and job arrangements may have to be tailored to their particular needs and remaining work capacity. This process requires close cooperation of all involved stakeholders, including patients and their families, employers, care providers, and insurers. It also has to address an interacting set of medical, vocational, demographic, psychological, and social factors [1–5]. In particular, many studies have stressed that psychosocial factors such as perception of health change, expectation of recovery, and social support are important predictors for successful RTW [6–10].

To address these issues and support RTW, various forms of case management have been introduced in industrialized countries for patients with psychiatric disorders or musculoskeletal injuries. Approaches differ widely with respect to target group and type of intervention provided [11–15].

The target group of our study are the victims of severe accidents insured at the Swiss National Accident Insurance Fund (Suva). In Switzerland, the total number of occupational and non-occupational accidents covered by compulsory accident insurance was more than 760,000 in 2008 [16]. Suva is the country’s largest accident insurance, with a market share of roughly 60 %. This high share is a result of regulations requiring industry, trade, commerce and construction companies to be mandatorily insured at Suva, as well as unemployed persons, state employees and many state-owned companies. Suva therefore covers 100 % of the accidents in these sectors.

Newly-registered accidents are segregated into procedural categories and handled by specialists applying means and methods tailored to each category. The most complex cases are those patients that are likely to suffer very long absence from work followed by substantial difficulties with vocational reintegration and an associated high risk of permanent disability. These patients take a disproportionate share of total insurance benefits. Support for these patients is provided by *claims specialists* and is here referred to as *standard case management (SCM)*.

A growing awareness of the difficulties associated with vocational reintegration, as well as steadily increasing insurance benefits for daily allowances and permanent disability pensions, has led the way to a new policy of providing intensive coaching to such patients. In 2002, a program was launched at Suva to establish *intensive case management (ICM)* provided by individually-assigned *case managers*. The program was intended for patients where more intensive coaching was assumed to improve healthcare treatment, support the patient’s rehabilitation process and RTW, and avoid a disability pension wherever possible.

Our study was tailored to the particular situation during the introductory years of ICM. Because adequate numbers of case managers had to be recruited and trained first, case manager resources in the early stages were not sufficient to meet the demand from eligible patients. Under these circumstances, it was ethically justifiable to allocate patients at random either to ICM or SCM, with the number of ICM cases limited by the availability of case managers. This transition phase was to end naturally at the time that available case manager resources came close to matching the demand. The aim of our study was to compare the effect of ICM (provided by case managers) and SCM (provided by claims specialists) on work incapacity and treatment costs.

## Methods

### Study Design

While our study uses an administrative dataset, the random procedure to allocate patients to alternative management strategies during a period of transition from one to another management strategy mimics a large randomized controlled trial.

Follow-up was intended to cover a period during which almost all patients would either have accomplished successful RTW or been allocated a permanent disability pension. As known from experience, a follow-up period of 6 years was required to achieve this goal. By then, the percentage of unresolved cases (patients still absent from work but not yet receiving a pension) would be so small that conclusions from the study could not be affected. Ethical approval was obtained from the Ethics Committee Northwestern and Central Switzerland under reference no. EKNZ-2015-008.

### Eligibility Criteria

Eligibility for randomization was limited to patients where medical complexity, difficulties with RTW and the risk of permanent disability were estimated to be serious enough to render ICM a valid option. This assessment was conducted by superior staff, based on their experience and expectation. Patients for whom intensive coaching was requested explicitly by themselves or their employer were excluded from the study and received this intervention outside it.

Random allocation to ICM or SCM started in 2002. To avoid bias from slight initial differences in implementation of the new process at local agencies, cases registered in 2002 were excluded from the study sample. The recruiting phase ended in 2006, because this was the last year with a large gap between demand and availability of case manager resources.

### Exclusion Criteria

For analysis, we excluded cases without coverage by the Swiss compulsory accident insurance. Furthermore, we excluded cases registered at Suva more than 12 months after the accident and patients with occupational diseases—although covered by compulsory accident insurance—because of limited comparability with injuries from accidents. Death during follow-up, whether as a direct consequence of the accident or not, was not grounds for exclusion, because fatalities were randomly distributed across study groups.

### Study Groups and Interventions

Both SCM and ICM are variants of what is sometimes referred to as *brokerage case management* [11, 15, 17–19], i.e. claims specialists and case managers administered and coordinated treatments from service providers, but did not provide medical or clinical treatment themselves. SCM and ICM differed primarily in the intensity of the assistance provided:

- The SCM group was treated according to Suva’s standard management procedure for severe accidents. Support is provided by very experienced *claims specialists*, with a caseload of approximately 100 cases. Assistance focuses on handling acute emerging problems and helping with RTW. The relationship to the patient usually does not include personal contact, as the patient is visited by field staff. The aim of SCM is to ensure that the patient receives the rehabilitation deemed necessary.
- The ICM group received more intensive and individually tailored coaching by specially trained *case managers*, with a typical caseload of 35 cases. The case manager’s responsibilities include assessing the patient’s needs, developing a care plan, providing personal assistance in all aspects of the rehabilitation and reintegration process, including coordination of healthcare treatment, monitoring the patient’s progress, and finding new work arrangements or helping to adapt existing ones. ICM encompasses a highly structured approach with defined steps:
  1. Establishing contact;
  2. Situation analysis in cooperation with consulting insurance physicians and other specialists;
  3. Planning of measures and defining objectives;
  4. Case management with clearly defined objectives, including personal contact and field visits to patients, employers and care providers; and
  5. Debriefing.

All activities are administered, coordinated and executed by the case manager. The focus is on satisfying patients’ needs, optimizing healthcare treatment and achieving the best occupational reintegration possible. The patient’s explicit agreement to cooperate closely with a personal case manager engaged by Suva was mandatory.

In both groups, patients are coached as long as considered appropriate by the responsible claims specialist or case manager respectively.

### Randomization Procedure

Randomization of eligible patients to SCM or ICM was based on a custom software with two operating modes, allowing for randomization of either a single patient (*N*_0_ = 1) or of a list of several patients (*N*_0_ ≥ 2). The ratio of allocation to SCM or ICM within each randomization step determines the weight of each case for statistical analysis (see “Appendix” for details).

### Primary Outcome Measure

Our study focuses on *average work incapacity* (AWI) as primary outcome measure. AWI denotes what percentage of the pre-accident work capacity was lost over the 6-year follow-up. This includes (1) short-term work incapacity, compensated by daily allowances, taken as percentage of pre-accident activity level; (2) permanent work incapacity compensated by disability pensions and calculated according to the degree of disability; (3) death as a consequence of the accident, counted as a 100 % work incapacity from the date of death. Disability pensions are substituting daily allowances as soon as work incapacity is regarded as permanent.

This concept of potential work capacity also holds for unemployed or part-time employed as well as for those who changed their employer after the accident or resumed work temporarily.

### Secondary Outcome Measures

We defined several secondary outcome measures:

- *Work incapacity* at a given reporting date, i.e. at the end of the n-th month after the accident (WI_n_, n = 12, 24, …, 72), expressed as a percentage of pre-accident work capacity. The definition is identical to AWI (see above), except that WI_n_ is a momentary snapshot while AWI is a 6-year average.
- *Disability pensions*, in CHF, paid for patients with permanent disability and graded according to the degree of disability. Typically, disability pensions are allocated several years after the accident.
- *Integrity indemnities*, in CHF, are one-off financial benefits for permanent damage to a person’s physical or mental integrity. The sum paid depends on the severity of the damage.
- *Treatment costs*, in CHF, are expressed as cumulative healthcare expenditure per patient, starting from the date of registration of the case until the end of each follow-up year. They include costs for healthcare treatment, medication, auxiliary material (bandages, implants, wheelchairs, etc.), rescue services, patient transport, and reintegration efforts (job recruiters). The proper costs for SCM and ICM (wages) are *not* included in treatment costs.
- *Number of care providers* involved in the treatment of patients over the entire follow-up period. This measure is based on information from our insurance claims database. We differentiate between independent physicians, insurance physicians, hospital out-patient, hospital in-patient, physiotherapists and ergotherapists, and other care providers. Claims specialists and case managers are not counted as care providers.
- *Length of stay in hospital* is the cumulative number of days spent as hospital in-patient.
- *Duration of coaching* is the number of months during which a patient was coached. This measure is only defined for case managers, but not for claims specialists.

### Independent Variables

Socio-demographic variables (such as gender, age class, civil status), occupational characteristics (flags for construction branch, employment status), and accident-related attributes (flags for sport, work or non-occupational accidents) were recorded at the date of registration of each accident.

The level of experience of a case manager at the time when he or she started coaching a new patient was approximated by the cumulative number of patients that he or she ever had coached up to that time. Experience levels were categorized into groups of 1–10, 11–20, 21–30, 31–50, or >50 patients.

### Statistical Analysis

Data analysis was generated using SAS software, Version 9.3 of the SAS System for Windows (SAS Institute Inc., Cary, NC, USA). Means, standard errors (SE) and statistical tests were calculated with the weights as derived from the randomization procedure (see “Appendix”).

To verify that the randomization procedure did not suffer from bias, independence of study groups was checked by Chi-square tests with regard to subgroups of patients defined by demographic, occupational and accident-related characteristics.

Outcomes between SCM and ICM were compared by *t* tests. Effect size (in percent) was calculated as outcome in ICM minus outcome in SCM, relative to outcome in SCM. To protect against the effects of non-normality on standard *t* tests, we used a nonparametric bootstrap procedure [20] with 5,000 Monte Carlo simulations to estimate two-sided *p* values and 95 % confidence intervals (CIs) for effect sizes. In the same way, we compared study groups in pre-specified subgroups as defined by independent variables.

Since we conducted a large number of statistical tests in this study, particularly for the subgroups analysis, we applied Bonferroni correction of individual *p* values to control the family wise error rate. Hence, an individual test should only be interpreted as statistically significant if its uncorrected *p* value, multiplied by the number of tests in the family, is < 0.05.

## Results

### Participant Flow

**Fig. 1:**
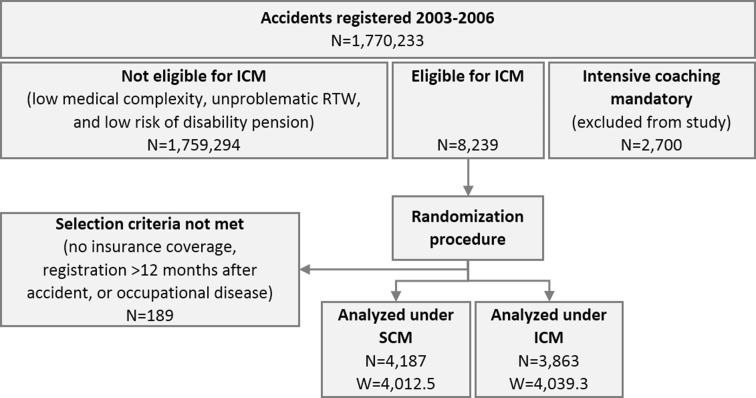
Participant flow with absolute (N) and weighted (W) number of cases. *SCM* standard case management, *ICM* intensive case management

Of the 8,239 patients eligible for coaching, the randomization mode for *N*_0_ = 1 was used for 1802 patients, of which 888 were allocated to SCM and 914 to ICM, and the mode for *N*_0_ ≥ 2 for 6,437 patients, of which 3,397 were allocated to SCM and 3040 to ICM (Fig. 1). The resulting mean weight was 1.05 (SD 0.22) for ICM and 0.96 (SD 0.10) for SCM. The minimum weight across all cases was 0.54, and only 5 cases had a weight >3.0, with a maximum of 6.5.

A total of 189 patients had to be excluded from analysis after randomization because they did not meet the selection criteria (Fig. 1). From the remaining 8,050 valid cases, 4,187 (weighted 4,012.5) were allocated to SCM and 3,863 (weighted 4,039.3) to ICM. Of the 4,187 patients allocated to SCM, 416 (weighted 401.5) were intensively coached because circumstances changed in a way that made this advisable. From the 3,863 patients allocated to ICM, 120 (weighted 142.5) never received intensive coaching because they proved to be less severe than initially estimated, or they declined coaching. We analyzed all cases according to their original treatment allocation (intention-to-treat principle [21]). Non-adherence to the original allocation would underestimate the differences between treatments.

The number of patients in subgroups defined by demographic, occupational and accident-related characteristics is presented in Table 1.

**Table 1:** Unweighted (N) and weighted (W) number of patients by demographic, occupational and accident-related characteristics. As expected, no significant differences were found with respect to the distribution of patients between these subgroups

	N (W)	SCM W (% of total)	ICM W (% of total)
Segmentations			
Total	8,050 (8,051.8)	4,012.5 (100.0 %)	4,039.3 (100.0 %)
Female	1,461 (1464.8)	723.2 (18.0 %)	741.6 (18.4 %)
Male	6,589 (6586.9)	3,289.3 (82.0 %)	3,297.6 (81.6 %)
Aged <30	1,934 (1933.4)	960.7 (23.9 %)	972.7 (24.1 %)
Aged 30–50	4,166 (4168.4)	2,061.2 (51.4 %)	2,107.2 (52.2 %)
Aged >50	1,950 (1949.9)	990.6 (24.7 %)	959.3 (23.7 %)
Married	4,446 (4452.9)	2,201.7 (54.9 %)	2,251.2 (55.7 %)
Unmarried	2,655 (2648.1)	1,352.5 (33.7 %)	1,295.6 (32.1 %)
Divorced	701 (702.6)	334.6 (8.3 %)	368.1 (9.1 %)
Other civil status	248 (248.0)	123.7 (3.1 %)	124.3 (3.1 %)
Occupational accident	2,983 (2971.0)	1,483.2 (37.0 %)	1,487.8 (36.8 %)
Non-occupational accident of employed person	4,543 (4554.4)	2,275.3 (56.7 %)	2,279.1 (56.4 %)
Accident of unemployed person	524 (526.4)	254.0 (6.3 %)	272.4 (6.7 %)
Flags			
Swiss nationality	5,009 (5000.6)	2,510.7 (62.6 %)	2,490.0 (61.6 %)
Commuters from neighbouring countries	615 (618.8)	299.4 (7.5 %)	319.5 (7.9 %)
Apprentice	367 (364.1)	181.5 (4.5 %)	182.6 (4.5 %)
Construction workers	1,267 (1269.4)	614.5 (15.3 %)	654.9 (16.2 %)
Temporary employment	466 (466.6)	223.0 (5.6 %)	243.7 (6.0 %)
Part time employment	704 (708.8)	340.3 (8.5 %)	368.6 (9.1 %)
Sport accident	1,190 (1193.2)	616.4 (15.4 %)	576.7 (14.3 %)

*SCM* standard case management, *ICM* intensive case management

### Duration of Coaching

The average duration of coaching in the ICM was 21.9 months (median 18). Only 169 patients (weighted 172.5) were still being coached at the end of the 6-year follow-up period. For the 416 patients originally allocated to SCM but then coached intensively, the average duration of coaching was 26 months. Only 30 (weighted 27.5) of these patients were still being coached at the end of the 6-year follow-up period.

### Work Incapacity

**Fig. 2:**
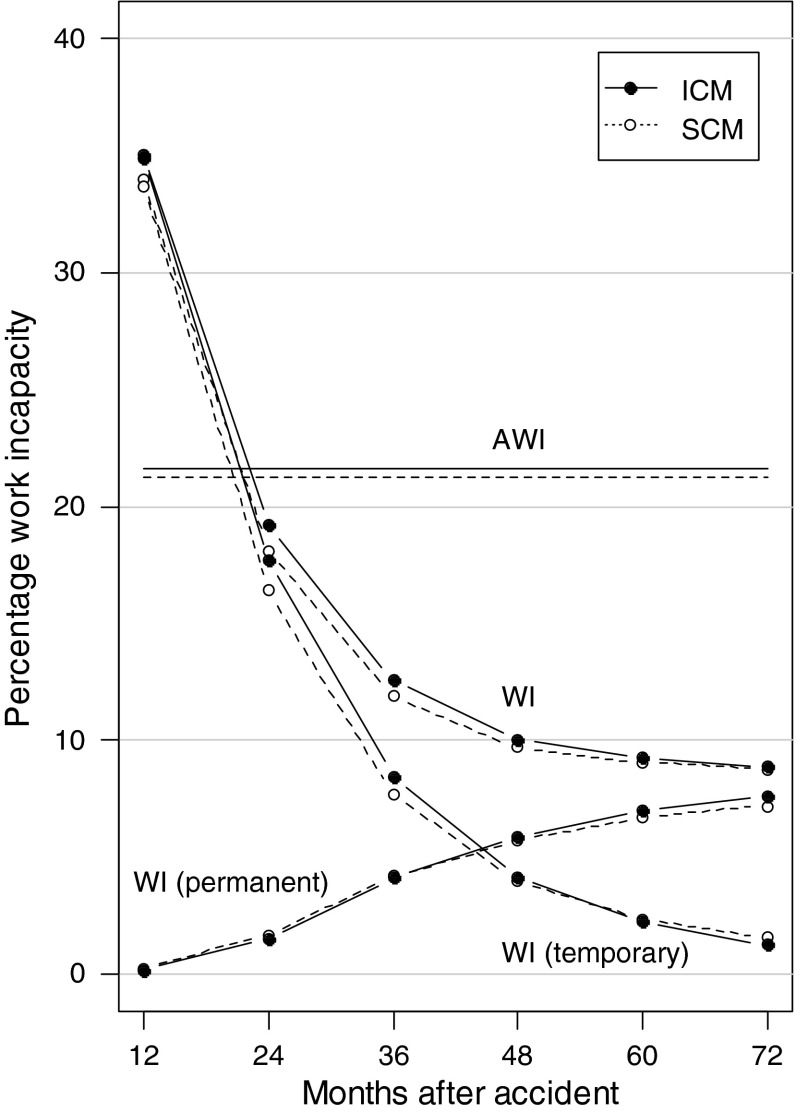
Work incapacity (WI) at the end of each follow-up year as a percentage of pre-accident work capacity. Contributions from permanent WI (permanent disability pensions and fatalities) and temporary WI (indemnified by daily allowances) are shown. *AWI* denotes daily work incapacity integrated over the entire 6-year follow-up period. *SCM* standard case management, *ICM* intensive case management

There was no difference between study groups with respect to absence from work. Work incapacity over the 6-year follow-up period is shown in Fig. 2, the endpoints in Table 2. Under SCM, work incapacity decreased from 34.0 % (SE 0.7) after 12 months to 8.8 % (SE 0.3) after 72 months, and under ICM from 35.1 % (SE 0.7) to 8.9 % (SE 0.4). None of these differences at intermediate measurements was statistically significant. At the end of the 6-year follow-up, contributions to work incapacity from permanent disability had reached 7.0 % under SCM and 7.4 % under ICM (*p* = 0.25), and the remaining work incapacity from patients that still received daily allowances but no permanent disability benefits yet was 1.6 % under SCM and 1.3 % under ICM (*p* = 0.14).

**Table 2:** Outcome variables at the end of the 6-year follow-up

	SCM Mean (SE)	ICM Mean (SE)	Effect (%) [95 % CI]	*p* value
Primary outcome				
Average work incapacity (AWI)				
In number of days lost from work	466 (8)	473 (8)	1.7 [−2.5–6.0]	0.44
In % of pre-accident work capacity	21.3 (0.36)	21.6 (0.38)		
Secondary outcomes				
Work incapacity at the end of 6-year follow-up (WI_72_), in % of pre-accident work capacity	8.8 (0.34)	8.9 (0.35)	0.9 [−8.0–10.9]	0.86
Thereof				
Indemnified by daily allowances	1.6 (0.18)	1.3 (0.17)	−20.7 [−42.0–7.7]	0.14
Indemnified by disability pension	7.0 (0.28)	7.4 (0.30)	6.2 [−3.7–17.5]	0.25
Due to fatalities	0.2 (0.07)	0.2 (0.07)	–7 [–48–130]	0.99
Disability pensions				
Percentage of patients with pensions	20.1 (0.62)	21.3 (0.66)	5.8 [−1.9–14.6]	0.16
Average degree (%) of disability for patients receiving a pension	34.7 (0.92)	34.9 (0.94)	0.4 [−6.0–7.2]	0.90
Treatment costs (in 1000 CHF)	39.8 (1.11)	43.5 (1.22)	9.4 [2.3–17.6]	0.01
Integrity indemnities				
Percentage of patients with indemnity	32.0 (0.72)	37.0 (0.78)	15.8 [9.5–22.2]	<0.001
Indemnities (in CHF) averaged across all patients in study group	6,996 (234)	7805 (248)	11.6 [2.8–21.1]	0.008
Indemnities (in CHF) averaged across patients receiving indemnities	21,881 (541)	21,089 (504)	−3.6 [−9.1–2.3]	0.24
Length of stay in hospital (number of days as in-patient)	28.9 (0.9)	30.7 (1.0)	6.3 [−0.1–13.0]	0.17

*SCM* standard case management, *ICM* intensive case management, *SE* standard error, *CI* confidence interval, *CHF* Swiss francs

AWI over the 6-year follow-up was 21.3 % under SCM and 21.6 % under ICM (*p* = 0.44). Both under SCM and ICM, about 70 % of the patients exhibited periods of partial work incapacity.

### Disability Pensions

We found no statistically significant differences in allocation of disability pensions between SCM and ICM (Table 2). The percentage of patients receiving permanent disability pensions at the end of the 6-year study period was 20.1 % under SCM and 21.3 % under ICM (*p* = 0.16). The average degree of disability across these patients was almost identical for both study groups (34.7 % under SCM vs. 34.9 % under ICM, *p* = 0.90). The study groups were also almost identical with respect to the point in time at which invalidity had been allocated: 61 % of the permanent pensions known at the end of the 6-year study period had been allocated in the first 36 months of the follow-up under SCM versus 60 % under ICM.

### Integrity Indemnities

As shown in Table 2, the percentage of patients who had received an integrity indemnity until the end of the 6-year follow-up was significantly higher under ICM (37.0 %) than under SCM (32.0 %, *p* < 0.001). However, for these patients, the average amount of indemnity paid was not significantly different under SCM (21,881 CHF) from ICM (21,089 CHF). Integrity indemnities were allocated at almost identical points in time: 67 % of the integrity indemnities known at the end of the 6-year study period had been allocated in the first 36 months of the follow-up under SCM versus 68 % under ICM.

### Treatment Costs

**Fig. 3:**
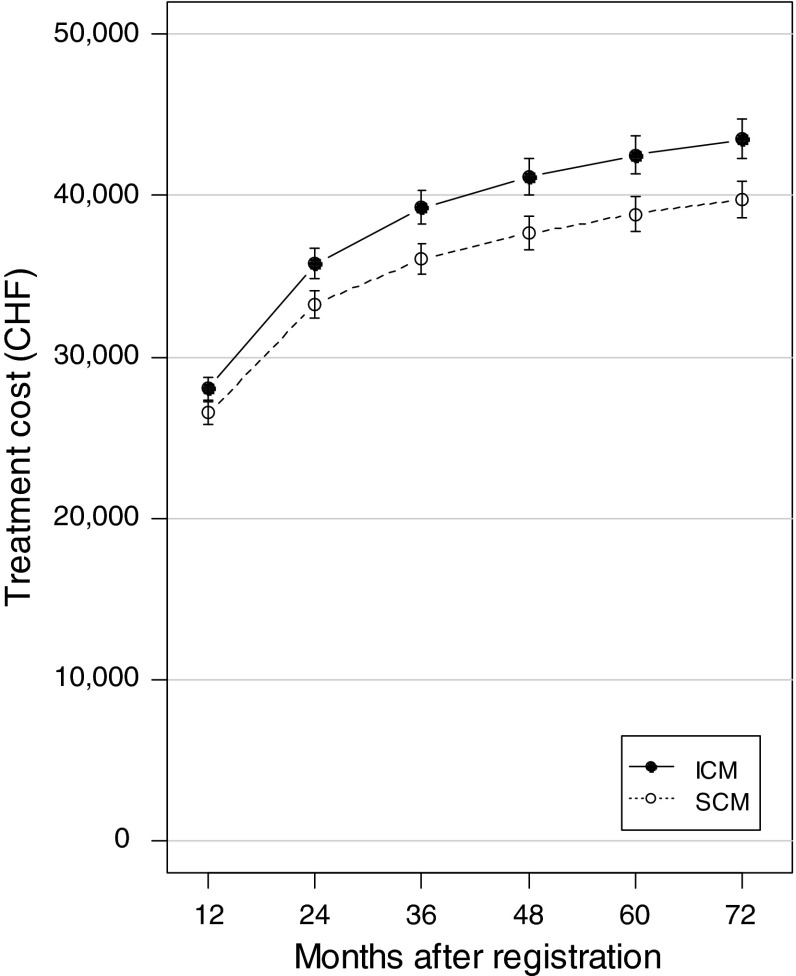
Treatment costs, accumulated from the date of registration of each case until the end of each follow-up year. *Error bars* are standard errors. *SCM* standard case management, *ICM* intensive case management

Throughout the 6-year follow-up period, average treatment costs per case were higher under ICM than under SCM. This difference increased over time and was statistically significant at each yearly measurement except for that at 12 months (Fig. 3; Table 2). At the end of the 6-year follow-up period, cumulative treatment costs were 39,800 CHF under SCM and 43,500 CHF under ICM (+9.4 %, *p* = 0.01).

### Care Providers

More care providers were involved when cases were processed under ICM as compared to SCM (Table 3). Relative effects ranged between 4.0 and 5.0 % at each yearly measurement (data not shown). At the end of the 6-year follow-up, there were on average 10.0 care providers involved in SCM cases vs. 10.5 in ICM (+5.0 %, *p* < 0.001). We found similar effects for all provider groups except for physiotherapists and ergotherapists. The biggest relative effect was found observed for insurance physicians (+8.3 % under ICM, *p* < 0.001).

**Table 3:** Number of care providers involved in treatment of patients and treatment costs over the 6-year follow-up period

Type of care provider	Number of care providers				Treatment costs (in 1000 CHF)			
	SCM Mean (SE)	ICM Mean (SE)	Effect (%) [95 % CI]	*p* value	SCM Mean (SE)	ICM Mean (SE)	Effect (%) [95 % CI]	*p* value
Total	10.00 (0.08)	10.50 (0.09)	5.0 [2.8–7.2]	<0.001	39.8 (1.11)	43.5 (1.22)	9.4 [2.0–17.2]	0.01
Independent physicians	2.70 (0.03)	2.79 (0.03)	3.3 [0.5–6.5]	0.03	3.0 (0.07)	3.1 (0.08)	3.9 [−2.3–10.4]	0.23
Insurance physicians	0.90 (0.01)	0.97 (0.01)	8.3 [5.5–11.1]	<0.001	0.95 (0.03)	1.04 (0.03)	8.9 [1.2–17.1]	0.02
Hospital out-patient	2.28 (0.03)	2.40 (0.03)	5.4 [2.3–8.7]	<0.001	4.8 (0.14)	5.4 (0.17)	13.7 [5.6–22.5]	0.001
Hospital in-patient	1.22 (0.02)	1.27 (0.02)	4.2 [0.8–7.7]	0.01	24.3 (0.86)	25.9 (0.92)	6.5 [−2.4–16.3]	0.17
Physiotherapists and ergotherapists	0.97 (0.01)	1.00 (0.01)	3.0 [−0.7–6.8]	0.11	2.2 (0.07)	2.2 (0.06)	0.4 [−7.3–8.5]	0.91
Other	1.95 (0.03)	2.08 (0.03)	6.9 [3.9–9.9]	<0.001	3.5 (0.17)	4.3 (0.19)	23.7 [13–36]	<0.001

Claims specialists and case managers are not included in these numbers. The ‘other’ group is a heterogeneous mixture of many different types of care providers and is therefore not interpretable

*SCM* standard case management, *ICM* intensive case management, *SE* standard error, *CI* confidence interval

We found significant differences in treatment costs at the end of the 6-year study period for insurance physicians (+8.9 % under ICM, *p* = 0.02) and for out-patient treatment in hospitals (+13.7 %, *p* = 0.001). However, at the end of the 6-year follow-up, there was no statistically significant difference with respect to length of stay in hospitals. Patients under SCM spent on average 28.9 days in hospitals and patients under ICM 30.7 days (+6.3 %, *p* = 0.17).

### Outcome by Subgroups

We analyzed differences between SCM and ICM for subgroups of patients defined by demographic, occupational and accident-related characteristics. We found no subgroups for which WI_72_ or AWI were significantly different between SCM and ICM (Table 4). A majority of subgroup comparisons had higher work incapacities under ICM than under SCM. We also observed lower values for ICM for patients aged under 30, divorced, with temporary occupations, or non-occupational accidents, but these effects were not significant. With respect to treatment costs, we found significantly higher values under ICM for several subgroups. However, after adjusting for multiple testing, only those for married patients were statistically significant.

**Table 4:** Outcomes by demographic, occupational and accident-related characteristics

	SCM Mean (SE)	ICM Mean (SE)	Effect (%) [95 % CI]	*p* value
Average work incapacity (AWI) in % of pre-accident work capacity				
Segmentations				
Total	21.3 (0.36)	21.6 (0.38)	1.7 [−2.5–6.3]	0.44
Female	16.2 (0.64)	17.5 (0.77)	8.1 [−3.2–20.3]	0.15
Male	22.4 (0.42)	22.6 (0.43)	0.8 [−4.0–5.7]	0.73
Aged <30	16.2 (0.64)	16.1 (0.64)	−0.9 [−10.2–9.5]	0.87
Aged 30–50	22.4 (0.52)	22.8 (0.53)	2.0 [−4.0–8.1]	0.53
Aged >50	23.9 (0.76)	24.8 (0.82)	3.5 [−4.5–12.3]	0.40
Married	23.8 (0.51)	24.7 (0.54)	3.7 [−1.7–9.5]	0.21
Unmarried	16.4 (0.54)	16.5 (0.55)	0.7 [−7.5–9.6]	0.88
Divorced	23.9 (1.33)	21.7 (1.23)	−9.1 [−21.2–5.0]	0.19
Other civil status	23.1 (2.23)	20.7 (2.07)	−10.5 [−30.6–14.5]	0.38
Occupational accident	24.9 (0.61)	26.2 (0.67)	5.2 [−1.4–12.2]	0.12
Non-occupational accident	19.0 (0.46)	18.7 (0.46)	−1.6 [−7.3–4.6]	0.62
Unemployed at time of accident	20.7 (1.42)	21.5 (1.43)	4.2 [−12.1–23.0]	0.65
Flags				
Swiss nationality	18.2 (0.41)	18.7 (0.44)	3.1 [−2.6–9.1]	0.31
Commuters from neighbouring countries	27.0 (1.51)	30.3 (1.58)	12.1 [−2.5–28.9]	0.11
Apprentice	12.5 (1.02)	12.7 (1.34)	1.9 [−19.4–28.8]	0.88
Construction workers	26.1 (0.97)	26.0 (1.02)	0.0 [−9.2–9.5]	0.99
Temporary employment	26.4 (1.60)	24.3 (1.61)	−7.8 [−21.9–8.2]	0.31
Part time employment	21.1 (1.29)	21.4 (1.27)	1.3 [−13.6–18.1]	0.87
Sport accident	13.3 (0.61)	14.3 (0.71)	7.3 [−4.7–20.3]	0.25
Work incapacity at the end of 6-year follow-up (WI_72_) in % of pre-accident work capacity				
Segmentations				
Total	8.8 (0.34)	8.9 (0.35)	0.9 [−8.5–11.5]	0.86
Female	3.9 (0.50)	5.3 (0.70)	36.0 [−2.3–88.5]	0.07
Male	9.9 (0.40)	9.7 (0.40)	−1.9 [−11.4–8.3]	0.70
Aged <30	5.1 (0.60)	4.3 (0.56)	−16.8 [−39.3–12.9]	0.25
Aged 30–50	9.2 (0.49)	9.3 (0.50)	0.5 [−12.1–15.0]	0.94
Aged >50	11.4 (0.71)	12.6 (0.79)	10.8 [−4.8–29.9]	0.20
Married	10.4 (0.48)	11.0 (0.51)	5.3 [−6.4–18.6]	0.38
Unmarried	5.5 (0.50)	5.5 (0.54)	−0.1 [−21.2–26.2]	0.97
Divorced	10.2 (1.25)	8.4 (1.09)	−17.6 [−40.1–13.2]	0.23
Other civil status	10.6 (2.19)	6.0 (1.69)	−43.1 [−71.3–4.4]	0.07
Occupational accident	10.8 (0.58)	12.0 (0.65)	11.1 [−3.0–27.1]	0.12
Non-occupational accident	7.7 (0.44)	6.9 (0.42)	−9.5 [−21.8–4.7]	0.18
Unemployed at time of accident	6.9 (1.25)	7.7 (1.31)	11.8 [− 28.2–77.1]	0.62
Flags				
Swiss nationality	7.0 (0.38)	7.3 (0.41)	3.9 [−9.7–20.0]	0.58
Commuters from neighbouring countries	11.0 (1.37)	13.9 (1.52)	25.6 [−6.1–69.5]	0.13
Apprentice	2.5 (0.92)	4.5 (1.46)	85.2 [−26.9–436.3]	0.18
Construction workers	11.1 (0.89)	13.3 (1.02)	19.8 [−1.8–46.0]	0.07
Temporary employment	10.2 (1.58)	8.9 (1.49)	−12.4 [−41.2–30.7]	0.51
Part time employment	10.4 (1.32)	9.8 (1.25)	−5.4 [−31.6–32.2]	0.74
Sport accident	3.7 (0.61)	4.2 (0.65)	11.8 [−24.8–65.5]	0.58
Treatment costs, in 1000 CHF				
Segmentations				
Total	39.8 (1.11)	43.5 (1.22)	9.4 [2.0–17.3]	0.01
Female	28.4 (1.50)	31.9 (1.99)	12.1 [−2.7–30.1]	0.13
Male	42.2 (1.31)	46.1 (1.42)	9.2 [1.0–18.2]	0.03
Aged <30	41.6 (2.59)	41.7 (2.55)	0.3 [−13.3–17.3]	0.97
Aged 30–50	39.8 (1.55)	44.8 (1.61)	12.4 [2.2–24.0]	0.02
Aged >50	37.9 (1.88)	42.6 (2.67)	12.4 [−2.7–29.7]	0.11
Married	37.9 (1.21)	43.7 (1.57)	15.4 [6.1–25.7]	0.001
Unmarried	42.0 (2.34)	43.3 (2.27)	3.2 [−9.5–18.2]	0.65
Divorced	42.5 (4.53)	45.1 (4.41)	6.3 [−17.5–37.1]	0.63
Other civil status	42.1 (6.24)	37.2 (5.31)	−11.8 [−39.1–29.0]	0.51
Occupational accident	39.7 (1.78)	46.2 (2.10)	16.3 [3.6–30.3]	0.01
Non-occupational accident	40.3 (1.51)	42.9 (1.63)	6.4 [−3.0–17.0]	0.16
Unemployed at time of accident	35.2 (4.28)	34.1 (2.86)	−3.2 [−25.6–26.2]	0.80
Flags				
Swiss nationality	39.3 (1.51)	43.0 (1.69)	9.4 [−0.5–20.4]	0.07
Commuters from neighbouring countries	43.2 (3.97)	55.3 (4.73)	28.2 [2.5–60.3]	0.03
Apprentice	39.8 (5.58)	51.1 (8.03)	28.6 [−12.2–86.0]	0.19
Construction workers	42.7 (2.61)	45.1 (2.66)	5.4 [−9.0–22.7]	0.49
Temporary employment	49.1 (5.50)	40.9 (3.56)	−16.7 [−34.4–7.9]	0.16
Part time employment	40.1 (4.19)	46.6 (4.30)	16.3 [− 9.3–48.5]	0.22
Sport accident	29.5 (2.10)	35.9 (3.61)	21.6 [−2.1–49.6]	0.08

*SCM* standard case management, *ICM* intensive case management, *SE* standard error, *CI* confidence interval

### The Effect of Case Managers’ Experience

For this particular subgroup analysis, we compared patients allocated to ICM only with patients that had been allocated to SCM in the *same* random drawing, with at least one patient allocated to each of the two study groups. Hence, we included only patients submitted to the randomization procedure using the mode for N_0_ ≥ 2 cases in this analysis.

A total of 5053 cases (weighted 5054.4) qualified for this analysis, 2667 under SCM (weighted 2531.6) and 2386 under ICM (weighted 2522.8). When case managers were very inexperienced (10 patients or fewer, Table 5), outcomes for AWI, WI_72_ and treatment costs were significantly higher under ICM than SCM. As experience increased, these differences diminished and for experienced case managers (>50 patients), outcomes approached those for the entire study sample.

**Table 5:** Effect of experience level of case managers on outcome

Cumulative number of patients coached per case manager	Effect (%) [95 % CI]	*p* value
Average work incapacity (AWI), in %		
Total	9.4 [3.6–15.7]	0.002
1–10	23.4 [10.0–38.6]	0.001
11–20	9.1 [−4.3–24.6]	0.19
21–30	8.2 [−6.7–25.4]	0.29
31–50	2.9 [−8.0–15.1]	0.62
≥51	3.7 [−7.6–16.5]	0.53
Work incapacity at the end of 6-year follow-up (WI_72_), in %		
Total	17.0 [3.3–32.7]	0.02
1–10	55.1 [20.8–100.9]	<0.001
11–20	22.5 [−8.1–63.7]	0.17
21–30	10.4 [−21.0–53.7]	0.55
31–50	2.8 [−20.7–33.3]	0.84
≥51	−3.9 [−27.9–29.0]	0.78
Treatment costs, in 1000 CHF		
Total	19.5 [9.4–30.5]	<0.001
1–10	44.1 [20.8–71.5]	<0.001
11–20	40.9 [13.1–74.8]	0.005
21–30	−4.1 [−21.7–17.8]	0.68
31–50	2.2 [−15.1–22.6]	0.81
≥51	15.7 [−3.1–37.9]	0.11

Experience level of a case manager is defined through the cumulative number of cases he or she has ever coached. Work incapacities are given as a percentage of pre-accident work capacity

*SE* standard error, *CI* confidence interval

## Discussion

We compared the effect of two competing forms of case management, ICM (provided by case managers) and SCM (provided by claims specialists), on work incapacity and treatment costs for patients who had suffered severe accidents.

### Work Incapacity and Disability Pensions

There was no statistically significant difference between SCM and ICM with respect to absence from work due to temporary (daily allowances) or permanent (disability pensions, fatalities) work incapacity. The only statistically significant effect with respect to permanent insurance benefits was found for the proportion of patients receiving integrity indemnities, which was higher under ICM (37 %) than under SCM (32 %).

This finding is contrary to expectations: The greater temporal resources that case managers were able to invest into patients’ rehabilitation and vocational reintegration had been expected to pay off in terms of faster RTW and/or lower disability pensions. This expectation was clearly not met. Our finding is also contrary to a considerable body of literature, where studies on patients with musculoskeletal disorders tend to report a reduction in time to RTW under various coordinating interventions [22–25]. However, a majority of these studies involved only a few hundred patients, and only a few studies exceeded one year of follow-up. Schandelmaier et al. [23] concluded that moderate quality evidence suggests limited effects of RTW coordination and that persistence and cost-effectiveness have yet to be confirmed in the long term. More in line with our findings are a number of studies that report absent or inconsistent intervention effects on RTW [26–28].

As to the reasons why ICM in our study was not superior to SCM with respect to RTW, we speculate that there may have been a tendency for case managers to prolong their efforts and ‘overcare’ for patients rather than to limit personal assistance to what is necessary under an economic maxim. This may also have been the consequence of a certain pressure for success felt by case managers based on expectations from peers and SCM claims specialists. It is therefore likely that case managers still pursued vocational reintegration efforts even when the probability for relevant improvements had become minimal. The more pragmatic and parsimonious approach of the claims specialists in the SCM group may therefore have been more efficient. This view is supported by our finding that the duration of coaching in the ICM group was highly correlated with AWI (Pearson correlation coefficient r = 0.59, *p* < 0.001). A literature review by Kuoppala and Lamminpää [29] concludes that any type of rehabilitation may have an effect at an early stage of decreased work ability, while becoming ineffective later on. However, we did not see such a temporal effect in our data as the difference between ICM and SCM was visible early in the study and persisted over the entire 6-year follow-up period.

Over the past 10 years, the absolute number of accidents that led to permanent disability pension claims to Suva continuously decreased [30]. The temporal coincidence with the introduction of ICM has been perceived as a causal relationship, but similar tendencies are observed in other parts of the Swiss social insurance system [31].

### Treatment Costs and Involved Care Providers

It is generally assumed that optimal coordination of medical therapies has a beneficial effect on rehabilitation and is therefore expected to reduce RTW. At Suva, rehabilitation efforts always focused on RTW rather than on treatment costs, and consequently case managers had not been instructed to minimize treatment costs. They were therefore likely to accept higher treatment costs wherever a concomitant reduction in RTW seemed possible.

Under these conditions, it is not a surprise that cumulative treatment costs were higher under ICM than under SCM throughout the follow-up period. The effect increased during follow-up and reached +9.4 % at the end. Along with the higher treatment costs, we observed a higher number of care providers (+5 %) involved in the treatment of patients under ICM. Both effects were strongest for Suva medical consultants and for hospital out-patient treatment, but weaker and not significant for hospital in-patient treatment. Support of Suva medical consultants is usually enlisted to get second opinions on medical questions or assessments of work incapacity wherever requested by patients, employers, or case managers. These findings demonstrate that case managers undertook various efforts to achieve optimal coordination of medical therapies, but eventually these efforts led to higher healthcare consumption.

More intensive coaching in our study was related to higher treatment costs. In contrast, a study on sick leave because of musculoskeletal disorders [32] reported significant savings in total costs under coordinated and tailored work rehabilitation, whereas (similar to our findings), savings in healthcare utilization costs were greatest for out-patient treatment, but not significant for in-patient treatment. Patients in the intervention group of that study also had more visits to a psychologist. Contrary to our findings, a study on the effect of integrated care on patients who were listed as sick for lower back pain reported a shorter duration until RTW [33] and a concomitant reduction in total treatment costs, and fewer consultations with general practitioners, therapists and psychologists [34].

### Outcome for Specific Subgroups

Despite our large sample size (implicating high statistical power), subgroups defined by demographic, occupational, or work-related characteristics were in general not significantly different between SCM and ICM. The only exception were higher treatment costs in ICM for married patients. However, we speculate that a slight though insignificant trend towards reduced work incapacity (WI_72_, AWI) under ICM may exist for the following subgroups:

- Patients with the most severe injuries, typically associated with prolonged work incapacity and very high insurance benefits, also have the highest variability of measured outcomes, and therefore the greatest potential for improvement. It might be sensible to focus intensive coaching on these patients.
- Patients with a weak social network or insufficient workplace integration such as divorced or widowed people, or temporary workers without steady employment, tended to have lower work incapacity under ICM than under SCM. On the other hand, patients in stable social networks (married, middle-aged, Swiss nationality, and/or permanent employment) tended to have higher work incapacity and treatment costs in ICM.

### Intensity and Duration of Coaching

Apart from the higher degree of formalization and broader decision-making competences, ICM differs from SCM mainly by a threefold lower caseload (approximately 35 vs. 100 cases). Caseload has been reported as an important factor affecting the success of case management, particularly in clinical settings with psychiatric patients. In these settings, caseloads also tend to be considerably lower than in our study. For example, in 16 out of 20 studies reviewed by Gorey et al. [35], case managers had caseloads of less than 20, and only four studies had caseloads of up to 40. The authors concluded that caseload was highly correlated with case management effect size. In a trial with severely mentally ill patients, caseload was 30–35 for standard case management and 10–15 for intensive case management [14, 36, 37]. However, these authors concluded that a lower caseload alone does not improve outcome for patients, and that the content of treatment may be more important than changes in service configurations. In our study, experience of case managers was an essential determinant of outcome, with outcomes rapidly improving as a consequence of learning. However, even very experienced case managers did not outperform claims specialists in the SCM group.

It is questionable whether conclusions from psychiatric settings can be transferred to case management for patients with musculoskeletal injuries. This is because studies with psychiatric patients typically address outcomes such as general behavior, social functioning, client and family satisfaction with services, or drop-out from services [38–40]. Such issues are ancillary in the context of accidents, because the focus is on physical rehabilitation. However, the patients’ well-being and satisfaction with case management may indirectly have beneficial effects on medical rehabilitation. At Suva, repeated surveys have consistently demonstrated higher satisfaction with services for patients that received intensive personal coaching than for those that did not (unpublished results). Similarly, Greenwood et al. [41] found that case management after severe head injury did not improve outcome; nonetheless the families of almost all patients who received intensive coaching were highly satisfied with the case manager. A study on integrated case management for work-related upper-extremity disorders found that intensive case management was significantly associated with greater patient satisfaction [42].

### Strengths and Limitations

The fact that our study is based entirely on administrative databases while still being in line with the concept of large randomized trials [43] gives it some outstanding features: The reported data are comprehensive, real life data with complete, long-term outcome measures. This is rarely encountered in the literature.

Our study also has some limitations:

1. Eligibility criteria for this study were based on the experience of the responsible superior staff, i.e. they were subjective to some degree. Furthermore, some patients were assigned directly to intensive coaching at their own or their employers’ request. Since we excluded these patients from the study, our study cases are not a random sample from the population of severe accidents registered at Suva, and generalizing the results requires caution.
2. Claims specialists in the SCM group in general were fully trained and experienced. In contrast, because our study was conducted in the initial years of the introduction of ICM, case managers in the ICM group were newly trained and had no experience with this new kind of coaching. Thus there is a significant difference between claims specialists and case managers in terms of professional experience. Personal characteristics, such as education or previous employment, were not recorded, hence we do not know whether they could have affected outcomes.
3. The insurer’s perception of the ICM approach as superior might have motivated claims specialists to adopt certain features of ICM over the years, triggering a moderate modernization of SCM. However, process variables collected during the study do not confirm a convergence of methods.
4. Our results solely reflect the vantage point of the insurance company and do not encompass total healthcare cost from a societal perspective. As we have no information about the patients’ occupational situation after payment of insurance benefits ceases, we cannot exclude the possibility that patients became unemployed or retired.

## Conclusions

Contrary to expectations, coaching patients under ICM did not reduce AWI or WI_n_ when compared to the situation under SCM. Instead, it led to significantly higher treatment costs and significantly more involved care providers. It seems that the intensity of case management alone is not sufficient to improve rehabilitation and vocational reintegration of severely injured patients from accidents.

## Appendix: Randomization Procedure

The software for randomizing patients to study groups was operated by submitting either a single patient (*N*_0_ = 1) or of a list of several patients (*N*_0_ ≥ 2) at the same time. The weight of each case for statistical analysis was then derived as follows:

### Operating Mode for *N*_0_ ≥ 2

A superior submitted a list of *N*_0_ eligible cases as well as the number *N*_CM_ of cases he wanted to assign to ICM, with the restriction that 1 ≤ *N*_CM_ ≤ *N*_0_/2. Free choice of *N*_CM_ had to be granted because there were limited resources for ICM. Because selection probability for ICM depended on the ratio *N*_CM_/*N*_0_, a compensating weighting scheme was required for statistical analysis. For the *N*_0_ cases on each list submitted to the randomization software, the cases’ individual weights *w*_i_ were calculated such that

$$ \mathop \sum \limits_{i = 1}^{{N_{0} }} w_{i} = N_{0} \quad {\text{for each list}} $$

and

$$ w_{\text{i}} = \raise.5ex\hbox{$\scriptstyle 1$}\kern-.1em/ \kern-.15em\lower.25ex\hbox{$\scriptstyle 2$} N_{0} /N_{\text{CM}} \quad {\text{for cases in ICM group}} $$

$$ w_{\text{i}} = \raise.5ex\hbox{$\scriptstyle 1$}\kern-.1em/ \kern-.15em\lower.25ex\hbox{$\scriptstyle 2$} N_{0} / \, \left( {N_{0} {-}N_{\text{CM}} } \right)\quad {\text{ for cases in SCM group,}} $$

thereby ensuring that the sum of the weights within a particular list was the same for both study groups.

### Operating Mode for *N*_0_ = 1

If a single case was submitted to the randomization procedure, it was allocated to either study group with a probability of 0.5. Therefore, the weight for these cases is *w*_i_ = 1.
